# Comprehensive Expression Profiling and Molecular Basis of CDC28 Protein Kinase Regulatory Subunit 2 in Cervical Cancer

**DOI:** 10.1155/2022/6084549

**Published:** 2022-07-28

**Authors:** Li Qin, Xiaoqiong Luo, Xiao Qin, Hongbao Huang, Lianling Zhang, Shengcai Chen, Xiaoqin Wu, Bingsheng Huang, Jian Pan, Jingxi Wei

**Affiliations:** ^1^Department of Obstetrics and Gynecology, The Affiliated Hospital of Youjiang Medical University for Nationalities, Baise, Guangxi Province 533000, China; ^2^Department of Obstetrics and Gynecology, People's Hospital of Baise, Baise, Guangxi Province 533000, China; ^3^Department of Pathology, The Affiliated Hospital of Youjiang Medical University for Nationalities, Baise, Guangxi Province 533000, China; ^4^Department of Human Anatomy, Guangxi Medical University, Nanning, Guangxi Province 530021, China

## Abstract

More and more evidence suggests the oncogenic function of overexpressed CDC28 protein kinase regulatory subunit 2 (CKS2) in various human cancers. However, *CKS2* has rarely been studied in cervical cancer. Herein, taking advantage of massive genetics data from multicenter RNA-seq and microarrays, we were the first group to perform tissue microarrays for CKS2 in cervical cancer. We were also the first to evaluate the clinical significance of *CKS2* with large samples (980 cervical cancer cases and 422 noncancer cases). We further excavated the mechanism of the tumor-promoting activities of *CKS2* in cervical cancer through analysis of genetic mutation profiles, Gene Ontology (GO), and Kyoto Encyclopedia of Genes and Genomes (KEGG) significant enrichment of genes coexpressed with *CKS2*. According to the results, expression data from multilevels unanimously supported the overexpression of *CKS2* in cervical cancer. Patients with cervical cancer in stage II from inhouse microarrays had significantly higher expression of *CKS2*, and *CKS2* overexpression had an adverse impact on the disease-free survival status of cervical cancer patients in GSE44001. Both mutation types of mRNA high and mRNA low appeared in cervical cancer cases from the TCGA Firehose project. Gene coexpressed with *CKS2* participated in pathways including the cell cycle, estrogen signaling pathway, and DNA replication. In summary, upregulated *CKS2* is closely associated with the malignant clinical development of cervical cancer and might serve as a valuable therapeutic target in cervical cancer.

## 1. Introduction

Cervical cancer is notorious as one of the most renowned malignant tumors in gynecology, with a high incidence in postmenopausal women [1]. There is a tendency for younger pathogenetic ages [2]. The latest statistics show that cervical cancer ranks as the fourth most common cancer in the worldwide female population, causing 604,127 newly diagnosed cases and 341,831 deaths in 2020 [3]. Multiple factors, such as premature sexual behavior, multiple personality partners, early marriage and early childbirth, multiple childbirth, smoking, and HIV infection, could raise the risk of cervical cancer [4]. Significant advances have been made in screening tests and treatment strategies, including chemotherapy and radiotherapy [5, 6]. An increasing amount of evidence suggests the benefits of the clinical application of a multidisciplinary approach to the management of female tumors, which helps improve the quality of life of patients [7–9]. However, metastasis or recurrence frequently occurs in a part of cervical cancer patients receiving the above treatment, and the prognosis of these patients is poor [10, 11]. Hence, looking for a new way of treatment has been the focus of long-term research in cervical cancer research.

CDC28 protein kinase regulatory subunit 2 (*CKS2*) is a member of the CKS family, which plays crucial roles in diverse biological activities and mediates the transition of cell cycles [12]. Increasing evidence suggests the oncogenic function of overexpressed *CKS2* in various human cancers, including adrenocortical carcinoma, tongue squamous cell carcinoma, lung adenocarcinoma, and hepatocellular carcinoma [13–16]. Transcriptomic analysis by Yang et al. revealed the significant prognostic value of *CKS2* for adrenocortical carcinoma [13]. Gao et al. reported that inhibiting *CKS2* expression in tongue squamous cell carcinoma could result in retarded cell growth and G2/M arrest in tumor cells [14]. The upregulation of *CKS2* in lung adenocarcinoma was related to worse survival of patients and larger tumor size [15]. Similarly, upregulated *CKS2* was observed to facilitate the malignant phenotype of hepatocellular carcinoma [16]. In light of the importance of *CKS2* as a hallmark for a broad type of tumor, it is worth investigating the clinicopathological significance and molecular mechanism of *CKS2* in cervical cancer.

*CKS2* has rarely been studied in cervical cancer. Therefore, we aimed to systematically appraise the clinicopathological significance and explore the molecular bases of *CKS2* in cervical cancer. For this, we used multiple detection technologies, including microarrays, RNA-seq, and immunohistochemistry (IHC).

## 2. Materials and Methods

### 2.1. Inhouse Tissue Microarray

In all, 124 cervical tissue samples were gathered by Panspectrum Biotechnology Limited Company, including 62 cervical cancer specimens and 62 noncancer cervix specimens (including mucosal inflammation and normal cervix tissues). Based on the judging criteria of the International Federation of Gynecology and Obstetrics, 31 cervical cancer patients were in stage I, and 31 cervical cancer patients were in stage II. Thirty-one cervical cancer patients were diagnosed with a T1 tumor according to the TNM staging published by the American Joint Committee on Cancer and Union International Center of Cancer. There was no distant metastasis or lymph node metastasis in any of the cases. All patients signed informed consent forms, and the ethics committee of the First Affiliated Hospital of Guangxi Medical University (approval ID: 2020(KY-E-095)) gave authority to the study.

The filtering of research objects, detailed procedures of IHC experiments, and the assessment rules of protein expression levels in tissue slides were described in an earlier study [17]. The antibody for CKS2 (https://www.abcam.cn/cks2-antibody-epr79462-ab155078.html) was used for incubation.

### 2.2. Additional Evidence from Other Microarrays and RNA-seq Datasets

Clinical data of cervical cancer patients and gene expression values (in the data format of fragments per kilobase per million or transcripts per kilobase million) in cervical cancer and noncancer cervix tissues were imported from The Cancer Genome Atlas (TCGA) database and the Genotype-Tissue Expression (GTEx) project. The incorporated dataset of the TCGA-GTEx expression matrix (containing 53 cervical adenocarcinomas, 253 cervical squamous cell carcinoma (CESC), and 14 noncancer samples) was normalized by the formula of log2 (transcripts per kilobase million value +0.001). Microarray datasets in the Gene Expression Omnibus (GEO) or ArrayExpress databases with a gene expression matrix of no less than three cervical cancers and three noncancer cervix samples belonging to Homo sapiens (before 11 June 2021) were other sources for expression analysis in the current study.

### 2.3. Comprehensive Expression Analysis for *CKS2* Utilizing Tissue Microarray, External RNA-seq, and Microarray Datasets

The extraction and processing of *CKS2* expression data were conducted according to the methods in prior work [18]. Microarrays were aggregated by the GPL platform, and the batch effect was removed for microarrays from the same platform through the limma package loaded with R software v. 3.6.1. Protein expression and diagnostic data of *CKS2* from the IHC scores of tissue microarrays were appended to the volume of other public microarray and RNA-seq datasets. The standard mean deviation (SMD) plot was calculated to comprehensively evaluate the different expressions of *CKS2* in cervical cancer versus noncancer tissues. The corresponding summarized receiver's operating characteristic (SROC) curves were painted. The steps of drawing SMD forest plots and SROC curves are found in previous studies [19].

### 2.4. Survival Analysis of *CKS2* in Cervical Cancer

The effect of *CKS2* expression on the prognostic situation of cervical cancer patients from the GSE44001 and RNA-seq datasets (overall survival plus disease-free survival) was estimated with Kaplan-Meier survival curves. Such curves were created with GEPIA and GraphPad Prism and a log-rank *P* value. The hazard ratio (HR) was calculated. The median *CKS2* expression value was the threshold for grouping patients, and *P* < 0.05 means statistical significance.

### 2.5. The Landscape of the Genetic Mutation of *CKS2* in Cervical Cancer

We used the cBioPortal database to examine mutation types and *z*-scores of mRNA expression (log RNA Seq V2 RSEM) of *CKS2* in 310 cervical cancer samples with mutation data from the GDAC Firehose project.

### 2.6. Characterization of the Molecular Function of *CKS2*-Coexpressed Genes in Cervical Cancer

We carried out differential expression analysis for the expression matrix of cervical cancer from all included microarrays with the limma package loaded in the R software v.3.6.1. Differentially expressed genes (DEGs) in the RNA-seq dataset were calculated with a count matrix from the voom algorithm in R software v. 3.6.1. DEGs of cervical cancer were genes that showed aberrant expression within cervical cancer and noncancer cervix specimens (log2FC > 1 or <-1 and adjusted *P* < 0.05) in no less than two datasets of RNA-seq or microarrays. The association between gene expression values was appraised through a Pearson correlation test embedded in the psych package loaded by R software v. 3.6.1. Upregulated DEGs that were positively correlated with the expression of *CKS2* (*r* > 0, adjusted *P* < 0.05) in one or more than one dataset of cervical cancer were regarded as genes positively related to *CKS2* in cervical cancer. Downregulated DEGs that had a negative relationship with *CKS2* (adjusted *P* < 0.05, *r* < 0) in one or more than one datasets of cervical cancer were defined as genes negatively correlated with *CKS2* in cervical cancer. Functional enrichment of the above genes with significant relationships to *CKS2* was annotated with the Kyoto Encyclopedia of Genes and Genomes (KEGG) pathway analysis and Gene Ontology (GO). Functional annotation was conducted with the ClusterProfiler package loaded in R software v. 3.6.1, and adjusted *P* < 0.05 was the cutoff value for significant KEGG and GO terms.

### 2.7. Statistical Analysis

SPSS 22.0 and GraphPad Prism v. 8.0.1 were applied to analyze inhouse tissue microarray data. At last, statistical significance was indicated by *P* < 0.05. An independent sample *t*-test was used to compare CKS2 expression in cervical cancer patients from tissue microarrays with different clinicopathological variables.

## 3. Results

### 3.1. CKS2 Expression in Cervical Cancer from Tissue Microarrays

**Figure 1:**
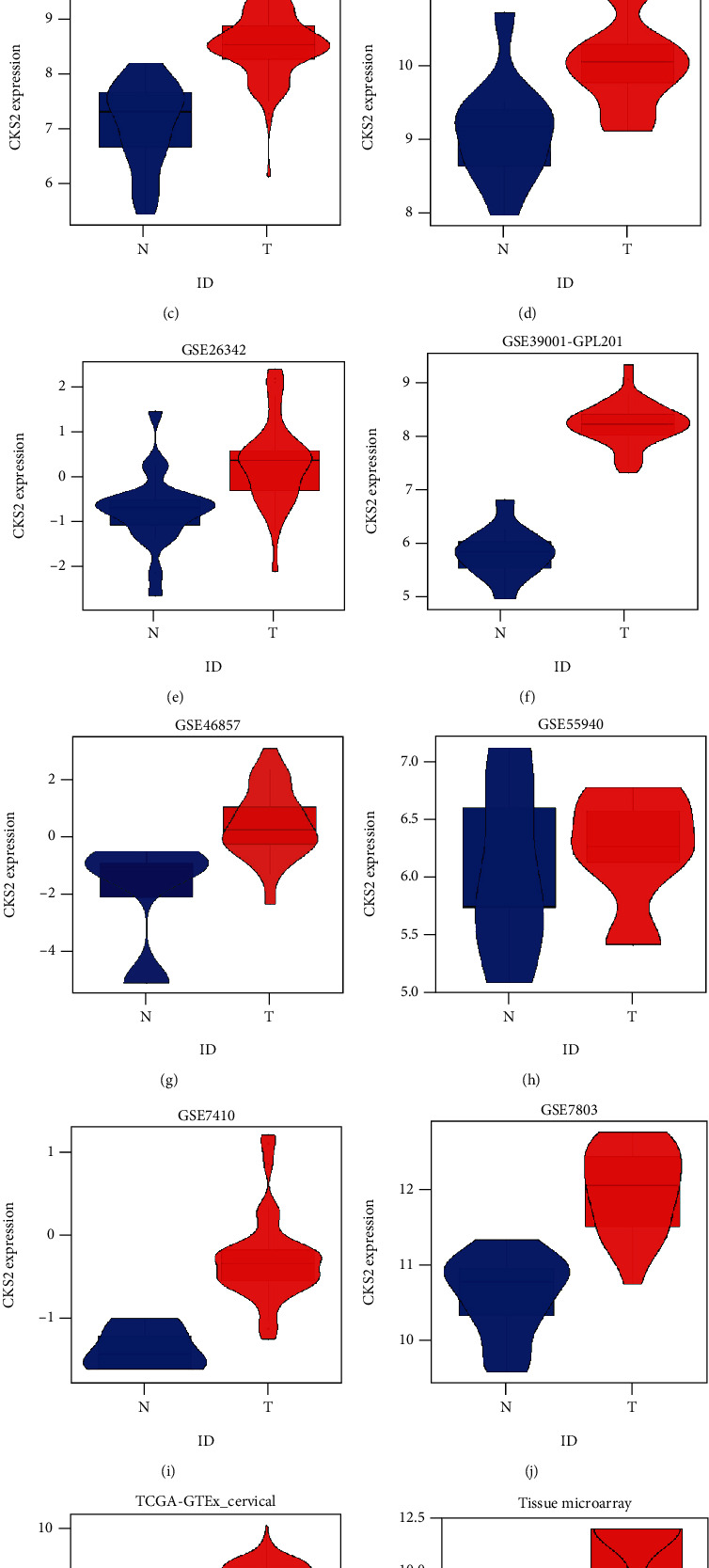
*CKS2* expression in cervical cancer and noncancer samples from inhouse tissue microarrays, external microarrays, and RNA-seq datasets. (a) Violin plots for GPL570. (b) Violin plots for GPL571. (c) Violin plots for GPL6244. (d) Violin plots for GPL96. (e) Violin plots for GSE138080. (f) Violin plots for GSE26342. (g) Violin plots for GSE39001 (GPL201). (h) Violin plots for GSE4482 (GPL3515). (i) Violin plots for GSE4482 (GPL4926). (j) Violin plots for GSE46857. (k) Violin plots for GSE55940. (l) Violin plots for GSE7410. (m) Violin plots for TCGA-GTEx datasets (n). Violin plots for inhouse microarray. The expression shown in blue is for individuals without cancer, and the expression shown in red is for individuals with cancer.

**Figure 2:**
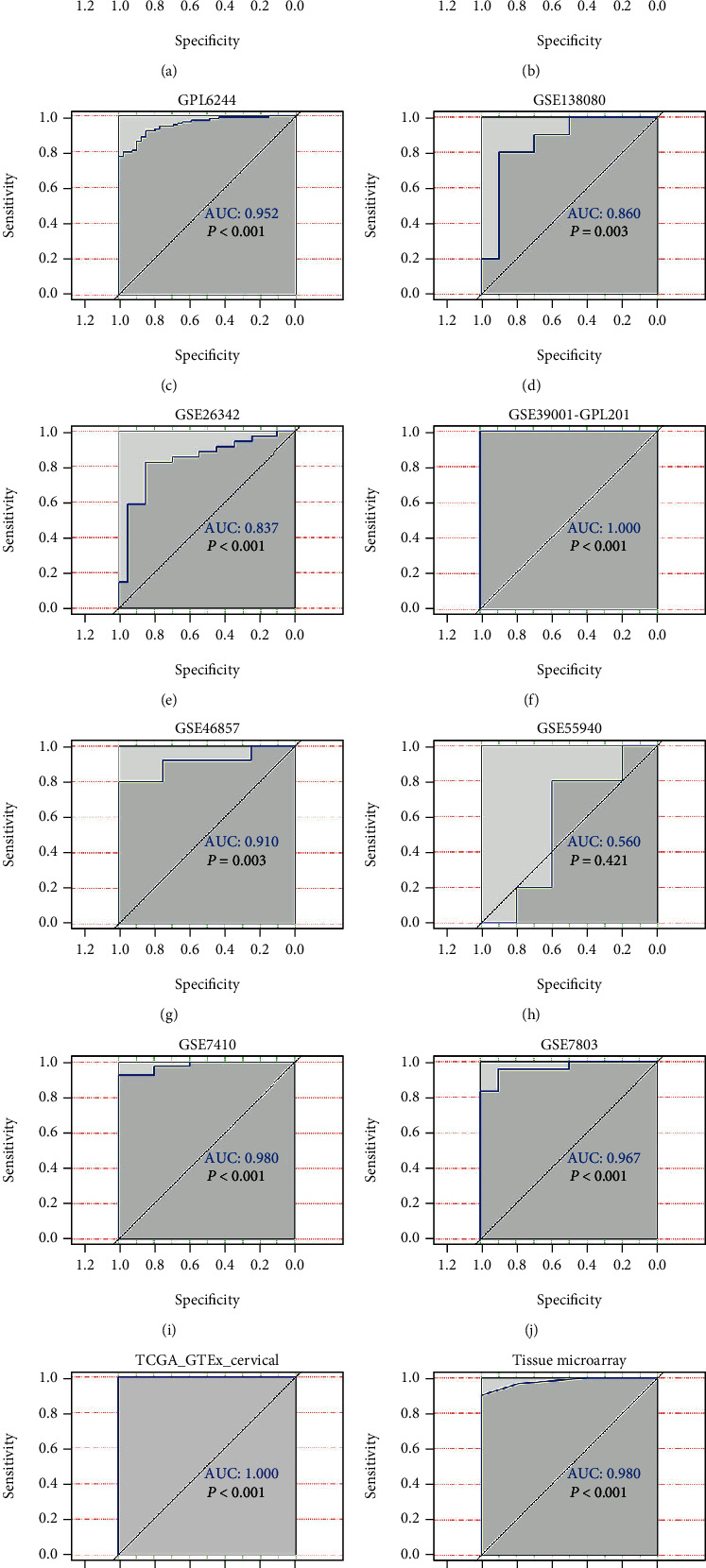
The discriminatory ability of *CKS2* expression in distinguishing cervical cancer from noncancer tissues in each microarray and RNA-seq dataset. (a) ROC curves for GPL570. (b) ROC curves for GPL571. (c) ROC curves for GPL6244. (d) ROC curves for GPL96. (e) ROC curves for GSE138080. (f) ROC curves for GSE26342. (g) ROC curves for GSE39001 (GPL201). (h) ROC curves for GSE4482 (GPL3515). (i) ROC curves for GSE4482 (GPL4926). (j) ROC curves for GSE46857. (k) ROC curves for GSE55940. (l) ROC curves for GSE7410. (m) ROC curves for TCGA-GTEx datasets. (n) ROC curves for inhouse microarray.

**Figure 3:**
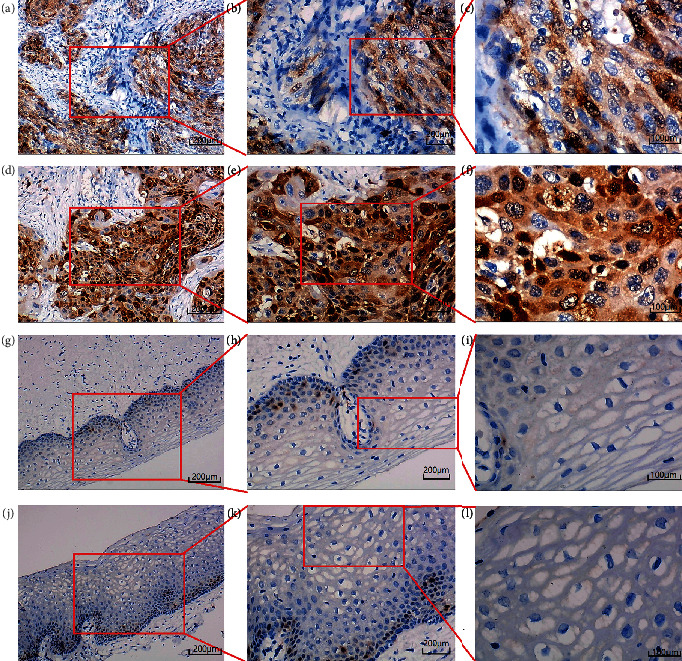
IHC staining of CKS2 in cervical cancer and noncancer tissues from tissue microarrays. (a) Moderate staining of CKS2 in cervical squamous cell carcinoma tissues (100x). (b) Moderate staining of CKS2 in cervical squamous cell carcinoma tissues (200x). (c) Moderate staining of CKS2 in cervical squamous cell carcinoma tissues (400x). (d) Strong staining of CKS2 in cervical squamous cell carcinoma tissues (100x). (e) Strong staining of CKS2 in cervical squamous cell carcinoma tissues (200x). (f) Strong staining of CKS2 in cervical squamous tissues (400x). (g) Negative staining of CKS2 in noncancer squamous epithelium tissues (100x). (h) Negative staining of CKS2 in noncancer squamous epithelium tissues (200x). (i) Negative staining of CKS2 in noncancer squamous epithelium tissues (400x). (j) Negative staining of CKS2 in noncancer squamous epithelium (100x). (k) Negative staining of CKS2 in noncancer squamous epithelium tissues (200x). (l) Negative staining of CKS2 in noncancer squamous epithelium tissues (400x).

**Figure 4:**
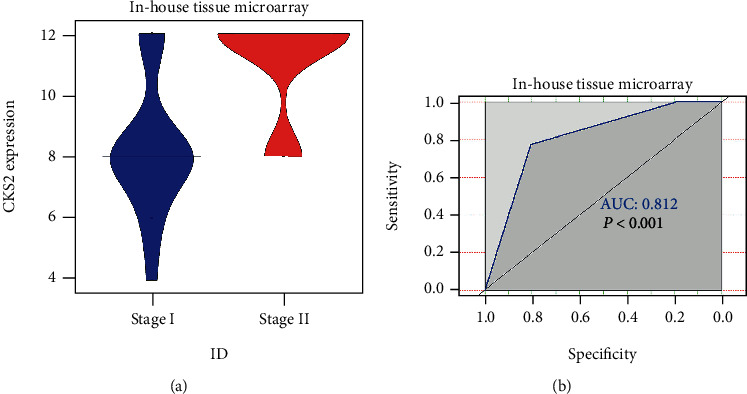
CKS2 expression in cervical cancer patients at different stages from tissue microarray. (a) Violin plot showing differential CKS2 expression between stage I and stage II groups. (b) ROC curves of CKS2 expression discriminate stage I cervical cancer patients from stage II cervical cancer patients.

CKS2 was significantly upregulated in 62 cervical cancer tissues in contrast to 62 noncancer tissues (*P* < 0.001) (9.677 ± 2.407; 3.629 ± 1.591) (Figures 1 and 2). IHC staining pictures reflected moderate or high immunoreactivity of CKS2 in the cancer nests of cervical cancer samples. In contrast, immunostaining of CKS2 exhibited negative or low reactivity in non-cancer cervix tissues (Figure 3). Moreover, CKS2 expression in cervical cancer patients diagnosed to be in clinical stage II (11.097 ± 1.700) was higher compared with that in cervical cancer patients with clinical stage I (8.258 ± 2.175) (*P* < 0.001) (Figure 4).

### 3.2. *CKS2* Overexpression in Cervical Cancer Samples from All Sources

**Figure 5:**
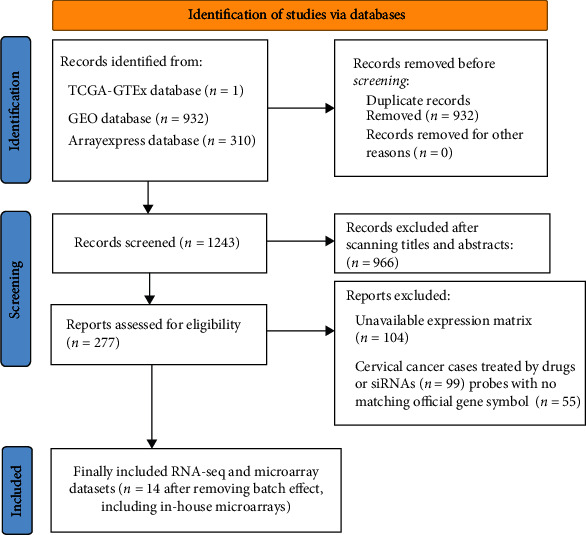
Flowchart of the inclusion of eligible microarrays and RNA-seq datasets for expression analysis.

**Figure 6:**
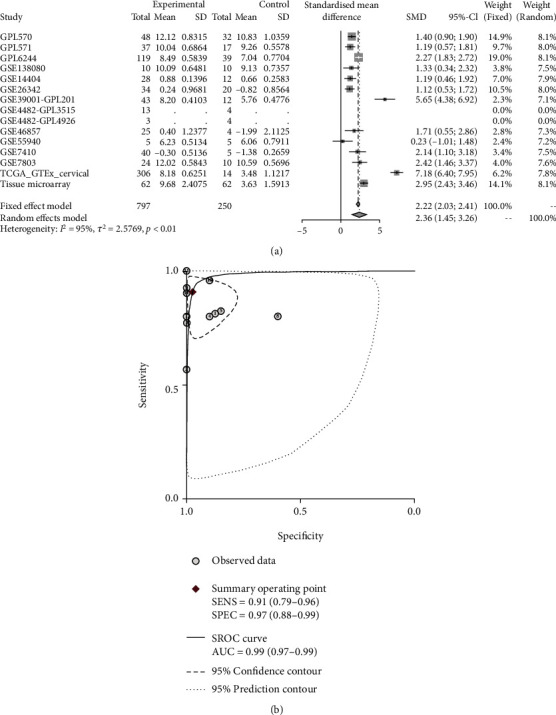
Pooled SMD forest plot and SROC curves of *CKS2* expression in cervical cancer and noncancer tissues for the inhouse tissue microarray, external microarrays, and RNA-seq datasets. (a) SMD forest. (b) SROC curves.

The process of screening qualified RNA-seq and microarrays for comprehensive expression analysis is depicted in Figure 5. There were 19 microarrays from the GEO database (14 microarrays after being merged by the GPL platform) included for comprehensive expression analysis. A summary of the basic elements of all included microarrays is listed in Table 1. Rich samples of 781 cervical cancers accompanied by 242 noncancer cases were collected from inhouse tissue microarray, other public RNA-seq, and microarrays. The remarkable high expression of *CKS2* in cervical cancer and the ability of *CKS2* overexpression to discriminate against cervical cancer and noncancer cervix tissues was revealed in most datasets (Figure 1). The overexpression of *CKS2* in cervical cancer was indicated in the forest plot of SMD. The SROC curves reflected the moderate capacity of *CKS2* overexpression in separating cervical cancer from noncancer cervix tissues (SMD = 2.36, 95%CI = 1.45–3.26; area under curve (AUC) = 0.99; Figure 6).

### 3.3. Prognostic Value of *CKS2* Expression for Cervical Cancer

**Figure 7:**
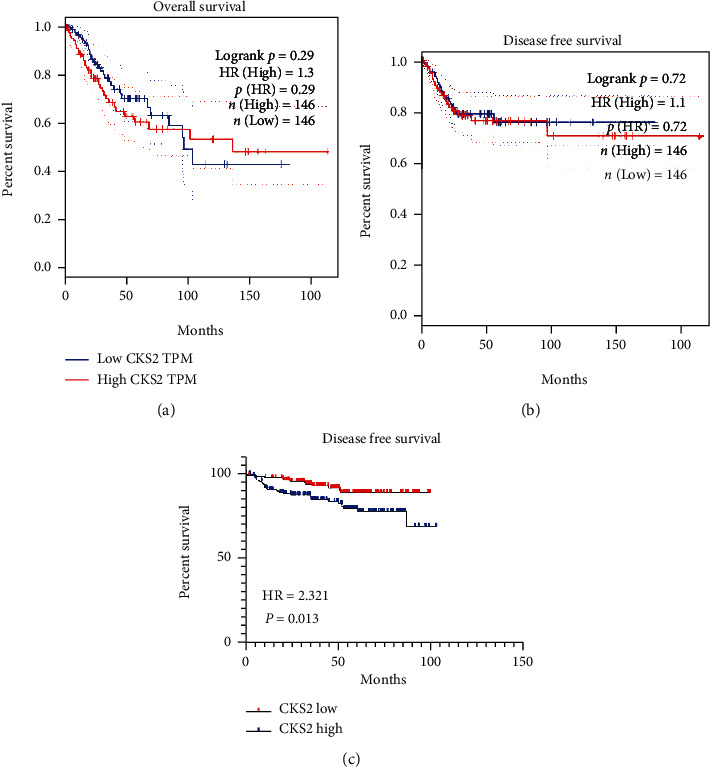
Survival analysis of *CKS2* expression in cervical cancer from the TCGA database and GSE44001. (a) Kaplan-Meier survival curves for overall survival of cervical cancer patients from the TCGA database. (b) Kaplan-Meier survival curves for disease-free survival of cervical cancer patients from the TCGA database. (c) Kaplan-Meier survival curves for disease-free survival of cervical cancer patients from GSE44001. TPM: transcripts per kilobase million; HR: hazard ratio.

The disease-free survival condition of cervical cancer patients (sampled from GSE44001) worsened in the group with *CKS2* high expression compared to cervical cancer patients with low *CKS2* expression (HR = 2.321, *P* = 0.013; Figure 7(c)). The prognostic results from the RNA-seq dataset were insignificant.

### 3.4. The Landscape of the Genetic Mutation Types of *CKS2* in Cervical Cancer

**Figure 8:**
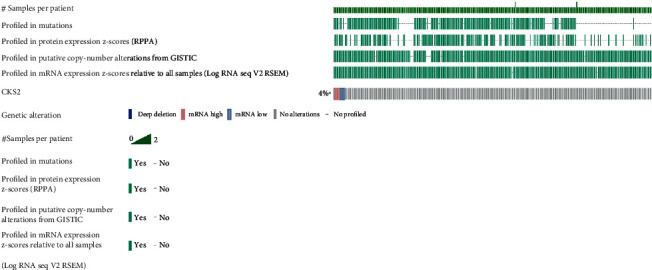
Genetic alteration of *CKS2* in cervical cancer. The bar chart demonstrates the genetic alteration status of *CKS2* in 310 cervical cancer samples profiled in mRNA expression and protein expression.

Five mRNA high cases, five with mRNA low, and one case of deep deletion were recorded in 310 cervical cancer cases from the TCGA database (Figure 8).

### 3.5. Diverse Functions of *CKS2*-Related Genes in Cervical Cancer

**Figure 9:**
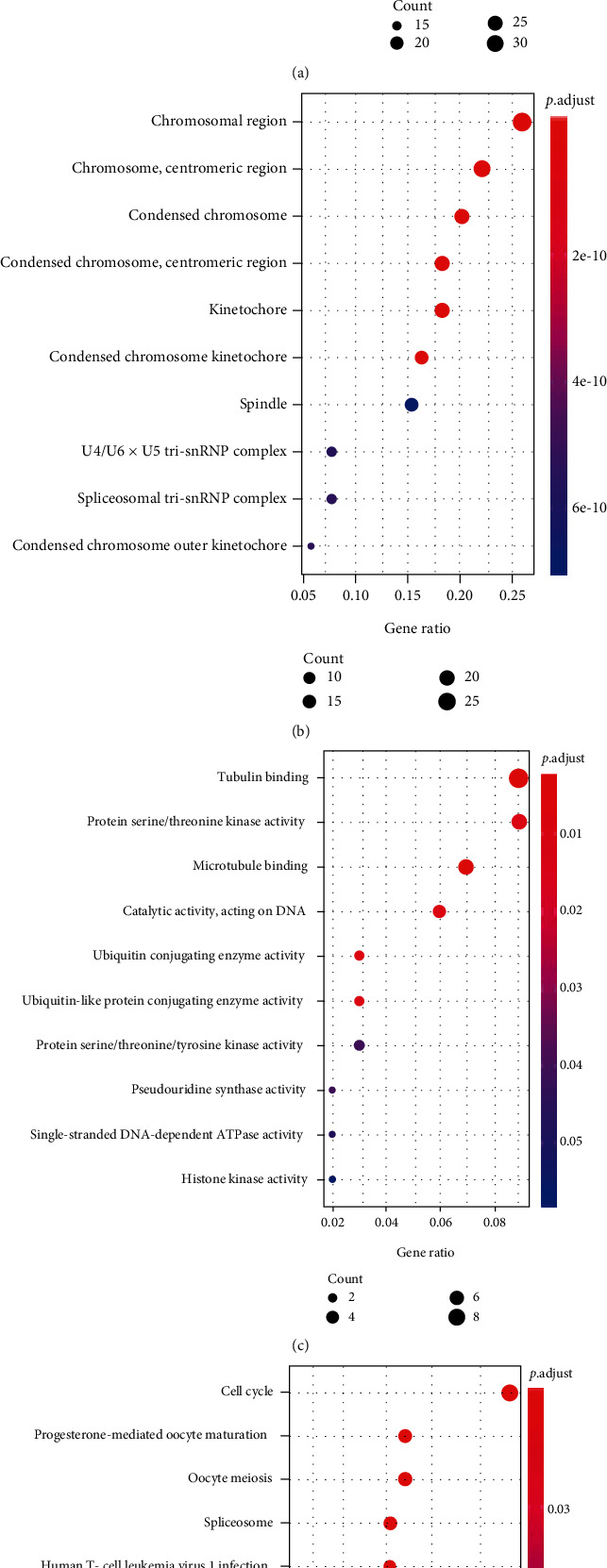
Functional enrichment analysis for genes positively correlated with *CKS2* in cervical cancer. (a) Dot plot for biological process terms. (b) Dot plot for cellular component terms. (c) Dot plot for molecular function terms. (d) Dot plot for pathway terms.

**Figure 10:**
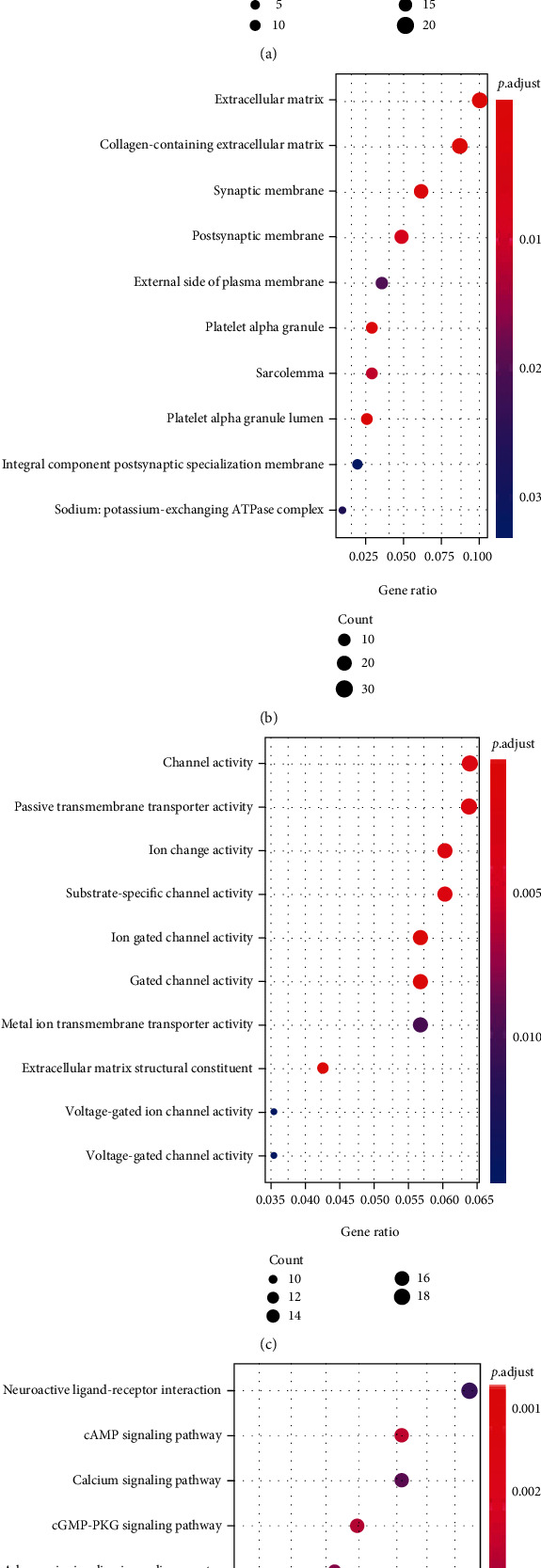
Functional enrichment analysis for genes negatively correlated with *CKS2* in cervical cancer. (a) Dot plot for biological process terms. (b) Dot plot for cellular component terms. (c) Dot plot for molecular function terms. (d) Dot plot for pathway terms.

RNA-seq data of cervical cancer in TCGA database and 22 external microarrays (e.g., GSE7803, GSE9750, GSE46857, GSE14404, GSE29570, GSE52903, GSE52904, GSE89657, GSE39001-GPL6244, GSE27678-GPL571, GSE63678, GSE6791, GSE27678-GPL570, GSE63514, GSE4482-GPL4926, GSE138080, GSE4482-GPL3515, GSE39001-GPL201, GSE7410, GSE55940, GSE67522, and GSE26342) were subjected to differential expression analysis and correlation analysis of gene expression. One hundred and four and 335 genes were positively and negatively related to *CKS2*, respectively (Supplementary Table 1). The engagement of these genes in various biological events and KEGG pathways is delineated in panels of dot plots (Figures 9 and 10).

## 4. Discussion

Big data produced from high-throughput RNA-seq and microarrays have been proven to be a powerful aid in oncology research, facilitating the hunting for novel biomarkers in a time-effective manner, which is how expression profiling and molecular investigations have improved the management of gynecological tumors [20–22]. Only two studies revealed the mitochondrial function of *CKS2* in the aggressive development of chemo radioresistant cervical cancer and the adverse impact of CKS2 high expression on the progression-free survival of cervical cancer patients [23, 24]. However, the clinicopathological significance and molecular basis of *CKS2* remain far from being expounded. Here, taking advantage of massive genetics data from multicenter microarrays and RNA-seq data, we evaluated the clinicopathological significance of *CKS2* with large samples (980 cervical cancers accompanied by 422 noncancer specimens). There is one precedent in history that excavated the potential mechanisms of the tumor-boosting activities of *CKS2* in cervical cancer.

Expression data from multilevels of inhouse tissue microarray, other public RNA-seq, and microarrays consistently supported the high expression of *CKS2* in cervical cancer. The expression analysis results from this study were convincing because of the large sample pool containing 980 cervical cancer plus 422 noncancer specimens. This phenomenon also agrees with the findings of earlier studies [23, 24]. Furthermore, the higher expression of *CKS2* in cervical cancer patients with stage II from inhouse microarrays and the adverse impact of *CKS2* overexpression on the disease-free survival conditions of cervical cancer patients in GSE44001 indicated the promotive effect of *CKS2* overexpression in augmenting the malignancy of cervical cancer.

After assessing the clinical significance of *CKS2* in cervical cancer, we further investigated the possible mechanisms of the tumor-boosting effect of *CKS2* in cervical cancer via genetic alteration analysis and functional enrichment annotation of genes co-expressed with *CKS2*. It could be noted from the bar chart of the alteration profile that both the mutation types of mRNA high and mRNA low appeared. Although the frequency of mRNA high and mRNA low equaled the genetic alteration profiles for the TCGA Firehose project, this only represented the mutation status of *CKS2* in the RNA-seq dataset. We conjectured that there might be a predominance of mRNA high over mRNA low in large samples. This includes cervical cancer specimens from microarrays and RNA-seq datasets, which might account for the upregulation of *CKS2* in cervical cancer. Apart from genetic mutation profile analysis, we also identified genes significantly related to *CKS2* in cervical cancer and their functional enrichment. There was an apparent difference between the terms of biological process, molecular function, and KEGG pathways assembled by genes positively and negatively related to *CKS2*. At the same time, plentiful biological process terms relevant to mitotic function could be found for genes positively associated with *CKS2*. The terms of extracellular constituent organization emerged in biological process and molecular function terms for genes negatively related to *CKS2*. Variation in the functional enrichment of genes positively and negatively associated with *CKS2* suggested that the interaction between *CKS2* and positively or negatively coexpressed genes might influence different aspects of biological function and molecular function in the initiation and development of cervical cancer. Concerning the KEGG pathway, multiple significantly assembled pathways, including the cell cycle, spliceosome, DNA replication, cellular senescence, MAPK signaling pathway, and estrogen signaling pathway, were closely linked with the carcinogenesis and pathophysiology of cervical cancer [25–38]. In particular, the involvement of *CKS2* in some significantly enriched pathways exampled by cell cycle and DNA replication has been reported by prior researchers [14, 39, 40]. Therefore, we postulated that the interrelationships between *CKS2* and genes related to *CKS2* might take part in cervical cancer oncogenesis through the above biological functions, molecular functions, and KEGG pathways.

The limitations of the present study were the lack of experimental validation of the functional roles of *CKS2* in cervical cancer and the connections between *CKS2* and other coexpressed genes, which should be warranted in future work.

## 5. Conclusion

In summary, *CKS2* was identified as being overexpressed in *CKS2* and is concerned with the clinical progression of cervical cancer. The oncogenic influence of *CKS2* overexpression in cervical cancer is related to the cell cycle, DNA replication, and estrogen signaling pathways. *CKS2* might serve as a valuable therapeutic target in cervical cancer.

## Figures and Tables

**Table 1:** Basic information from all included RNA-seq and microarray datasets of cervical cancer.

Dataset	Platform	Country	First author	Sample type	Number of tumor samples	Number of noncancer samples
GSE7803	GPL96	USA	Rork Kuick	Tissue	24	10
GSE46857	GPL7025	India	Rita Mulherkar	Tissue	25	4
GSE14404	GPL6699	India	Rajkumar T	Tissue	28	12
GSE29570	GPL6244	Mexico	Mariano Guardado-Estrada	Tissue	119	39
GSE52903	GPL6244	Mexico	Ingrid Medina Martinez	Tissue		
GSE52904	GPL6244	Mexico	Ingrid Medina Martinez	Tissue		
GSE39001	GPL6244	Mexico	Ana María Espinosa	Tissue		
GSE27678	GPL571	United Kingdom	Ian Roberts	Tissue and cell lines	37	17
GSE63678	GPL571	USA	Prokopios Alexandros Polyzos	Tissue		
GSE6791	GPL570	USA	Paul Ahlquist	Tissue	48	32
GSE63514	GPL570	USA	Johan den Boon	Tissue		
GSE4482	GPL4926	India	Chandan Kumar	Tissue	3	4
GSE138080	GPL4133	Netherlands	Renske DM Steenbergen	Tissue	10	10
GSE4482	GPL3515	India	Chandan Kumar	Tissue	13	4
GSE39001	GPL201	Mexico	Ana María Espinosa	Tissue	43	12
GSE7410	GPL1708	Netherlands	Petra Biewenga	Tissue	40	5
GSE55940	GPL16238	China	Chen Ye	Tissue	5	5
GSE67522	GPL10558	United Kingdom	Sweta Sharma Saha	Tissue	20	22
GSE26342	GPL1053/GPL1052	USA	Natalia Shulzhenko	Tissue	34	20
TCGA-GTEx	—	—	—	Tissue	306	14

## Data Availability

The datasets generated and/or analyzed during the current study are available in the TCGA (https://portal.gdc.cancer.gov/) and GEO (https://www.ncbi.nlm.nih.gov/gds) databases.

## Supplementary Materials

Supplementary Materials Supplementary Table 1: genes positively and negatively related to CKS2.
