# Supplementary material for: Comprehensive Expression Profiling and Molecular Basis of CDC28 Protein Kinase Regulatory Subunit 2 in Cervical Cancer
Source: Int J Genomics. 2022 Jul 28;2022:6084549. doi: 10.1155/2022/6084549 (PMC9352497; doi:10.1155/2022/6084549)
Supplement: Supplementary Materials — Supplementary Table 1: genes positively and negatively related to CKS2. [file 6084549.f1.docx]

Supplementary Table 1. Genes positively and negatively related to CKS2

| ID | Classification |
| --- | --- |
| CCNB1 | positively CKS2 related genes |
| BIRC5 | positively CKS2 related genes |
| CDC20 | positively CKS2 related genes |
| KPNA2 | positively CKS2 related genes |
| CENPA | positively CKS2 related genes |
| EXOSC3 | positively CKS2 related genes |
| CDKN3 | positively CKS2 related genes |
| CDCA5 | positively CKS2 related genes |
| NDUFA8 | positively CKS2 related genes |
| PTTG1 | positively CKS2 related genes |
| SNRPD1 | positively CKS2 related genes |
| NCAPG | positively CKS2 related genes |
| PSRC1 | positively CKS2 related genes |
| CDCA3 | positively CKS2 related genes |
| MND1 | positively CKS2 related genes |
| UBE2C | positively CKS2 related genes |
| PBK | positively CKS2 related genes |
| PSMB7 | positively CKS2 related genes |
| SPC25 | positively CKS2 related genes |
| RAN | positively CKS2 related genes |
| POLE3 | positively CKS2 related genes |
| TRUB2 | positively CKS2 related genes |
| NUP37 | positively CKS2 related genes |
| SNRPF | positively CKS2 related genes |
| PLK1 | positively CKS2 related genes |
| AURKB | positively CKS2 related genes |
| RANBP1 | positively CKS2 related genes |
| MAD2L1 | positively CKS2 related genes |
| FANCC | positively CKS2 related genes |
| ZMYND19 | positively CKS2 related genes |
| ORC6 | positively CKS2 related genes |
| SKA1 | positively CKS2 related genes |
| UBE2S | positively CKS2 related genes |
| LYAR | positively CKS2 related genes |
| SHMT2 | positively CKS2 related genes |
| NDC80 | positively CKS2 related genes |
| BUB1 | positively CKS2 related genes |
| PIF1 | positively CKS2 related genes |
| CCNB2 | positively CKS2 related genes |
| CCNA2 | positively CKS2 related genes |
| SNRPG | positively CKS2 related genes |
| BUB3 | positively CKS2 related genes |
| AURKA | positively CKS2 related genes |
| MELK | positively CKS2 related genes |
| DEPDC1 | positively CKS2 related genes |
| SPAG5 | positively CKS2 related genes |
| UBE2T | positively CKS2 related genes |
| CACYBP | positively CKS2 related genes |
| DLGAP5 | positively CKS2 related genes |
| OIP5 | positively CKS2 related genes |
| TSFM | positively CKS2 related genes |
| MRPL13 | positively CKS2 related genes |
| CDK1 | positively CKS2 related genes |
| CENPN | positively CKS2 related genes |
| HJURP | positively CKS2 related genes |
| SPC24 | positively CKS2 related genes |
| THOP1 | positively CKS2 related genes |
| RACGAP1 | positively CKS2 related genes |
| FANCG | positively CKS2 related genes |
| PRPF4 | positively CKS2 related genes |
| CDC45 | positively CKS2 related genes |
| KIF2C | positively CKS2 related genes |
| KIF4A | positively CKS2 related genes |
| SAC3D1 | positively CKS2 related genes |
| PAICS | positively CKS2 related genes |
| HSPE1 | positively CKS2 related genes |
| POLD2 | positively CKS2 related genes |
| LMNB2 | positively CKS2 related genes |
| CDC25A | positively CKS2 related genes |
| MRTO4 | positively CKS2 related genes |
| MIF | positively CKS2 related genes |
| NCBP1 | positively CKS2 related genes |
| CDCA8 | positively CKS2 related genes |
| KIF20A | positively CKS2 related genes |
| DDX39A | positively CKS2 related genes |
| SNRPB | positively CKS2 related genes |
| LSM4 | positively CKS2 related genes |
| FEN1 | positively CKS2 related genes |
| AIMP2 | positively CKS2 related genes |
| DEPDC1B | positively CKS2 related genes |
| NDUFB9 | positively CKS2 related genes |
| DBF4 | positively CKS2 related genes |
| EBNA1BP2 | positively CKS2 related genes |
| TROAP | positively CKS2 related genes |
| CDC25C | positively CKS2 related genes |
| CCT6A | positively CKS2 related genes |
| NCAPH | positively CKS2 related genes |
| PPIH | positively CKS2 related genes |
| CKS1B | positively CKS2 related genes |
| NCLN | positively CKS2 related genes |
| LSM5 | positively CKS2 related genes |
| PSAT1 | positively CKS2 related genes |
| PSMB5 | positively CKS2 related genes |
| TTK | positively CKS2 related genes |
| FAM72B | positively CKS2 related genes |
| HYLS1 | positively CKS2 related genes |
| KIFC1 | positively CKS2 related genes |
| POLR2D | positively CKS2 related genes |
| TIMM8A | positively CKS2 related genes |
| DSCC1 | positively CKS2 related genes |
| NME1 | positively CKS2 related genes |
| DKC1 | positively CKS2 related genes |
| NEIL3 | positively CKS2 related genes |
| BUB1B | positively CKS2 related genes |
| NR0B1 | negatively CKS2 related genes |
| PLEKHH2 | negatively CKS2 related genes |
| GP6 | negatively CKS2 related genes |
| MMP19 | negatively CKS2 related genes |
| ECHDC2 | negatively CKS2 related genes |
| FAM110B | negatively CKS2 related genes |
| TMEM220 | negatively CKS2 related genes |
| SLC7A2 | negatively CKS2 related genes |
| CTSK | negatively CKS2 related genes |
| ADCY2 | negatively CKS2 related genes |
| SMPDL3A | negatively CKS2 related genes |
| EPHA5 | negatively CKS2 related genes |
| PCDHB4 | negatively CKS2 related genes |
| A2M | negatively CKS2 related genes |
| CTTNBP2 | negatively CKS2 related genes |
| RASSF2 | negatively CKS2 related genes |
| GPR20 | negatively CKS2 related genes |
| DEFB124 | negatively CKS2 related genes |
| CPXM2 | negatively CKS2 related genes |
| CCDC80 | negatively CKS2 related genes |
| CHAD | negatively CKS2 related genes |
| KALRN | negatively CKS2 related genes |
| NDN | negatively CKS2 related genes |
| PGR | negatively CKS2 related genes |
| MYL3 | negatively CKS2 related genes |
| ADAMTS9 | negatively CKS2 related genes |
| RGS5 | negatively CKS2 related genes |
| ZNF208 | negatively CKS2 related genes |
| LHFPL3 | negatively CKS2 related genes |
| PDZRN3 | negatively CKS2 related genes |
| ACSM5 | negatively CKS2 related genes |
| PTH1R | negatively CKS2 related genes |
| ISLR | negatively CKS2 related genes |
| IDUA | negatively CKS2 related genes |
| DOK6 | negatively CKS2 related genes |
| CD177 | negatively CKS2 related genes |
| PNPLA7 | negatively CKS2 related genes |
| LMO2 | negatively CKS2 related genes |
| IGSF9B | negatively CKS2 related genes |
| LRRN4CL | negatively CKS2 related genes |
| EDN3 | negatively CKS2 related genes |
| PCP4 | negatively CKS2 related genes |
| RASGRP2 | negatively CKS2 related genes |
| C6 | negatively CKS2 related genes |
| LUM | negatively CKS2 related genes |
| CHST8 | negatively CKS2 related genes |
| ARHGAP6 | negatively CKS2 related genes |
| RAPGEF3 | negatively CKS2 related genes |
| NPY5R | negatively CKS2 related genes |
| FXYD1 | negatively CKS2 related genes |
| MEF2C | negatively CKS2 related genes |
| GEM | negatively CKS2 related genes |
| ANKRD20A5P | negatively CKS2 related genes |
| RHBDF1 | negatively CKS2 related genes |
| NAP1L2 | negatively CKS2 related genes |
| CLCF1 | negatively CKS2 related genes |
| ADH4 | negatively CKS2 related genes |
| PRLR | negatively CKS2 related genes |
| PEG3 | negatively CKS2 related genes |
| TNMD | negatively CKS2 related genes |
| FMOD | negatively CKS2 related genes |
| SLC5A4 | negatively CKS2 related genes |
| LMOD1 | negatively CKS2 related genes |
| SLC46A2 | negatively CKS2 related genes |
| ALDH1A1 | negatively CKS2 related genes |
| PRDM1 | negatively CKS2 related genes |
| FOS | negatively CKS2 related genes |
| SIGLEC6 | negatively CKS2 related genes |
| FCER1A | negatively CKS2 related genes |
| SLC15A1 | negatively CKS2 related genes |
| FGA | negatively CKS2 related genes |
| GRID1 | negatively CKS2 related genes |
| PENK | negatively CKS2 related genes |
| PDGFD | negatively CKS2 related genes |
| SLITRK3 | negatively CKS2 related genes |
| QPRT | negatively CKS2 related genes |
| YPEL5 | negatively CKS2 related genes |
| BAZ2B | negatively CKS2 related genes |
| SPATA6 | negatively CKS2 related genes |
| CACNA1G | negatively CKS2 related genes |
| FYCO1 | negatively CKS2 related genes |
| GYPC | negatively CKS2 related genes |
| HSPB7 | negatively CKS2 related genes |
| PTGER2 | negatively CKS2 related genes |
| FAM124B | negatively CKS2 related genes |
| TRHDE | negatively CKS2 related genes |
| KCNIP1 | negatively CKS2 related genes |
| GRM7 | negatively CKS2 related genes |
| REG1A | negatively CKS2 related genes |
| S1PR1 | negatively CKS2 related genes |
| CACNA1H | negatively CKS2 related genes |
| SPINK5 | negatively CKS2 related genes |
| THBS3 | negatively CKS2 related genes |
| IGF2 | negatively CKS2 related genes |
| SCGB3A1 | negatively CKS2 related genes |
| FAM107A | negatively CKS2 related genes |
| SH3BGRL2 | negatively CKS2 related genes |
| KIAA0408 | negatively CKS2 related genes |
| NAP1L3 | negatively CKS2 related genes |
| ISLR2 | negatively CKS2 related genes |
| FAIM2 | negatively CKS2 related genes |
| LRRC2 | negatively CKS2 related genes |
| C9orf106 | negatively CKS2 related genes |
| SYNE1 | negatively CKS2 related genes |
| CRYM | negatively CKS2 related genes |
| UBL3 | negatively CKS2 related genes |
| LDOC1 | negatively CKS2 related genes |
| PODN | negatively CKS2 related genes |
| PPP1R3C | negatively CKS2 related genes |
| CREB3L2 | negatively CKS2 related genes |
| TCEAL2 | negatively CKS2 related genes |
| PCDHB15 | negatively CKS2 related genes |
| RPRM | negatively CKS2 related genes |
| SSPN | negatively CKS2 related genes |
| CHRD | negatively CKS2 related genes |
| CLU | negatively CKS2 related genes |
| DIO3 | negatively CKS2 related genes |
| ZNF676 | negatively CKS2 related genes |
| EPB41L3 | negatively CKS2 related genes |
| MAP6 | negatively CKS2 related genes |
| GAS7 | negatively CKS2 related genes |
| RARRES2 | negatively CKS2 related genes |
| C1orf21 | negatively CKS2 related genes |
| TPSG1 | negatively CKS2 related genes |
| GSTM5 | negatively CKS2 related genes |
| MYOC | negatively CKS2 related genes |
| CMAHP | negatively CKS2 related genes |
| FAM20A | negatively CKS2 related genes |
| LCN6 | negatively CKS2 related genes |
| DOK5 | negatively CKS2 related genes |
| JAM2 | negatively CKS2 related genes |
| CPA3 | negatively CKS2 related genes |
| RORB | negatively CKS2 related genes |
| RIMBP2 | negatively CKS2 related genes |
| CLEC4F | negatively CKS2 related genes |
| CD69 | negatively CKS2 related genes |
| KCNK3 | negatively CKS2 related genes |
| RGCC | negatively CKS2 related genes |
| CYP2A6 | negatively CKS2 related genes |
| ALDH1A2 | negatively CKS2 related genes |
| YPEL2 | negatively CKS2 related genes |
| GFRA2 | negatively CKS2 related genes |
| FREM1 | negatively CKS2 related genes |
| CPXM1 | negatively CKS2 related genes |
| RERG | negatively CKS2 related genes |
| HDC | negatively CKS2 related genes |
| EBF1 | negatively CKS2 related genes |
| TWIST2 | negatively CKS2 related genes |
| LAMA2 | negatively CKS2 related genes |
| MMP2 | negatively CKS2 related genes |
| CADPS | negatively CKS2 related genes |
| CPZ | negatively CKS2 related genes |
| SORBS2 | negatively CKS2 related genes |
| EMCN | negatively CKS2 related genes |
| FAM131B | negatively CKS2 related genes |
| FBLN5 | negatively CKS2 related genes |
| SLC18A2 | negatively CKS2 related genes |
| ZEB1 | negatively CKS2 related genes |
| RAI2 | negatively CKS2 related genes |
| CXCL12 | negatively CKS2 related genes |
| HCG22 | negatively CKS2 related genes |
| ASXL3 | negatively CKS2 related genes |
| SNTG2 | negatively CKS2 related genes |
| SFRP1 | negatively CKS2 related genes |
| NDRG2 | negatively CKS2 related genes |
| CPED1 | negatively CKS2 related genes |
| ZNF185 | negatively CKS2 related genes |
| EPB41L4A | negatively CKS2 related genes |
| ACADL | negatively CKS2 related genes |
| MRVI1 | negatively CKS2 related genes |
| METTL7A | negatively CKS2 related genes |
| SCN7A | negatively CKS2 related genes |
| ASPA | negatively CKS2 related genes |
| PLCXD3 | negatively CKS2 related genes |
| PRRG3 | negatively CKS2 related genes |
| SNCAIP | negatively CKS2 related genes |
| ARHGAP27 | negatively CKS2 related genes |
| MRGPRF | negatively CKS2 related genes |
| SCNN1D | negatively CKS2 related genes |
| NTRK3 | negatively CKS2 related genes |
| RNASE4 | negatively CKS2 related genes |
| MROH7 | negatively CKS2 related genes |
| H6PD | negatively CKS2 related genes |
| IGSF5 | negatively CKS2 related genes |
| ADCY4 | negatively CKS2 related genes |
| SCNN1G | negatively CKS2 related genes |
| CLCA4 | negatively CKS2 related genes |
| ACSM1 | negatively CKS2 related genes |
| CCDC181 | negatively CKS2 related genes |
| SLC2A10 | negatively CKS2 related genes |
| HTR2B | negatively CKS2 related genes |
| LONRF2 | negatively CKS2 related genes |
| LRRC4C | negatively CKS2 related genes |
| DIO2 | negatively CKS2 related genes |
| ANK2 | negatively CKS2 related genes |
| GDPD3 | negatively CKS2 related genes |
| F7 | negatively CKS2 related genes |
| ATP1B2 | negatively CKS2 related genes |
| TSPYL5 | negatively CKS2 related genes |
| SLC7A3 | negatively CKS2 related genes |
| DUSP1 | negatively CKS2 related genes |
| FMO2 | negatively CKS2 related genes |
| CA10 | negatively CKS2 related genes |
| GPRASP1 | negatively CKS2 related genes |
| MYO15B | negatively CKS2 related genes |
| SLITRK4 | negatively CKS2 related genes |
| ANKRD35 | negatively CKS2 related genes |
| CH25H | negatively CKS2 related genes |
| LEFTY2 | negatively CKS2 related genes |
| CYS1 | negatively CKS2 related genes |
| ESR1 | negatively CKS2 related genes |
| TRH | negatively CKS2 related genes |
| NYNRIN | negatively CKS2 related genes |
| CCDC33 | negatively CKS2 related genes |
| ABI3BP | negatively CKS2 related genes |
| HAND2 | negatively CKS2 related genes |
| TPCN1 | negatively CKS2 related genes |
| HPGD | negatively CKS2 related genes |
| CDH19 | negatively CKS2 related genes |
| CCDC178 | negatively CKS2 related genes |
| RSPO1 | negatively CKS2 related genes |
| EDNRB | negatively CKS2 related genes |
| LRRK2 | negatively CKS2 related genes |
| XYLT1 | negatively CKS2 related genes |
| GFRA1 | negatively CKS2 related genes |
| CCDC170 | negatively CKS2 related genes |
| SPNS2 | negatively CKS2 related genes |
| FRZB | negatively CKS2 related genes |
| KCNA2 | negatively CKS2 related genes |
| FRMD4B | negatively CKS2 related genes |
| ADRA1D | negatively CKS2 related genes |
| SORCS1 | negatively CKS2 related genes |
| TMEM130 | negatively CKS2 related genes |
| CSDC2 | negatively CKS2 related genes |
| MAN1C1 | negatively CKS2 related genes |
| HPSE2 | negatively CKS2 related genes |
| FOSB | negatively CKS2 related genes |
| LSAMP | negatively CKS2 related genes |
| PAMR1 | negatively CKS2 related genes |
| F13A1 | negatively CKS2 related genes |
| PER1 | negatively CKS2 related genes |
| PROK1 | negatively CKS2 related genes |
| CLIP3 | negatively CKS2 related genes |
| LYNX1 | negatively CKS2 related genes |
| AQP1 | negatively CKS2 related genes |
| ZNF454 | negatively CKS2 related genes |
| IL34 | negatively CKS2 related genes |
| GALNT13 | negatively CKS2 related genes |
| PIK3C2B | negatively CKS2 related genes |
| SLC5A9 | negatively CKS2 related genes |
| DNAH1 | negatively CKS2 related genes |
| MS4A2 | negatively CKS2 related genes |
| TSC22D3 | negatively CKS2 related genes |
| TPSAB1 | negatively CKS2 related genes |
| IL1R1 | negatively CKS2 related genes |
| APOD | negatively CKS2 related genes |
| C7 | negatively CKS2 related genes |
| CREB3L1 | negatively CKS2 related genes |
| GNG7 | negatively CKS2 related genes |
| ADAM33 | negatively CKS2 related genes |
| FGD5 | negatively CKS2 related genes |
| KRT4 | negatively CKS2 related genes |
| GRIN2A | negatively CKS2 related genes |
| RPS6KA6 | negatively CKS2 related genes |
| PRSS27 | negatively CKS2 related genes |
| EMX2OS | negatively CKS2 related genes |
| NRXN2 | negatively CKS2 related genes |
| TCEAL5 | negatively CKS2 related genes |
| LDLRAD4 | negatively CKS2 related genes |
| RBPMS | negatively CKS2 related genes |
| PDGFRA | negatively CKS2 related genes |
| KCND3 | negatively CKS2 related genes |
| BHLHE22 | negatively CKS2 related genes |
| NR3C2 | negatively CKS2 related genes |
| LRRC4B | negatively CKS2 related genes |
| HAND2-AS1 | negatively CKS2 related genes |
| NBEA | negatively CKS2 related genes |
| LRFN5 | negatively CKS2 related genes |
| CYP2B7P | negatively CKS2 related genes |
| HNMT | negatively CKS2 related genes |
| FGF7 | negatively CKS2 related genes |
| PLAC9 | negatively CKS2 related genes |
| DPT | negatively CKS2 related genes |
| COL14A1 | negatively CKS2 related genes |
| ABCB1 | negatively CKS2 related genes |
| AFF3 | negatively CKS2 related genes |
| C1QTNF7 | negatively CKS2 related genes |
| ATP1A2 | negatively CKS2 related genes |
| PTGDS | negatively CKS2 related genes |
| ABCA8 | negatively CKS2 related genes |
| F10 | negatively CKS2 related genes |
| NUDT10 | negatively CKS2 related genes |
| ANXA9 | negatively CKS2 related genes |
| PTPRN2 | negatively CKS2 related genes |
| CXCR2 | negatively CKS2 related genes |
| CNTN4 | negatively CKS2 related genes |
| ZFP36 | negatively CKS2 related genes |
| CYP4F12 | negatively CKS2 related genes |
| PARM1 | negatively CKS2 related genes |
| ACKR1 | negatively CKS2 related genes |
| GABRB3 | negatively CKS2 related genes |
| ABCA6 | negatively CKS2 related genes |
| CRISPLD2 | negatively CKS2 related genes |
| NOVA1 | negatively CKS2 related genes |
| SCNN1B | negatively CKS2 related genes |
| STEAP4 | negatively CKS2 related genes |
| ZBTB16 | negatively CKS2 related genes |
| BPI | negatively CKS2 related genes |
| TMEM132C | negatively CKS2 related genes |
| KSR1 | negatively CKS2 related genes |
| MMRN1 | negatively CKS2 related genes |
| ITGA8 | negatively CKS2 related genes |
| SYTL5 | negatively CKS2 related genes |
| MFAP4 | negatively CKS2 related genes |
| CRISP3 | negatively CKS2 related genes |
| CHL1 | negatively CKS2 related genes |
| SFRP4 | negatively CKS2 related genes |
| MASP1 | negatively CKS2 related genes |
| SNED1 | negatively CKS2 related genes |
| KCNK15 | negatively CKS2 related genes |
| RHOU | negatively CKS2 related genes |
| ZNF385D | negatively CKS2 related genes |
| VIPR2 | negatively CKS2 related genes |
| ADRA2A | negatively CKS2 related genes |
| ARHGEF10L | negatively CKS2 related genes |
| CACNA1C | negatively CKS2 related genes |
| CEACAM7 | negatively CKS2 related genes |
| SLC24A3 | negatively CKS2 related genes |
| SELP | negatively CKS2 related genes |
| FAM3D | negatively CKS2 related genes |
| CAPN5 | negatively CKS2 related genes |
| PLBD1 | negatively CKS2 related genes |
| CCDC69 | negatively CKS2 related genes |
| CYP3A5 | negatively CKS2 related genes |
| BCAS1 | negatively CKS2 related genes |
